# Optimal timing of preoperative indocyanine green administration for fluorescent cholangiography during laparoscopic cholecystectomy using the PINPOINT® Endoscopic Fluorescence Imaging System

**DOI:** 10.1111/ases.12440

**Published:** 2017-12-19

**Authors:** Nobuhiro Tsutsui, Masashi Yoshida, Hikaru Nakagawa, Eisaku Ito, Ryota Iwase, Norihiko Suzuki, Tomonori Imakita, Hironori Ohdaira, Masaki Kitajima, Katsuhiko Yanaga, Yutaka Suzuki

**Affiliations:** ^1^ Department of Surgery International University of Health and Welfare Hospital Nasushiobara Japan; ^2^ Department of Surgery Jikei University School of Medicine Tokyo Japan

**Keywords:** Fluorescent cholangiography, laparoscopic cholecystectomy, PINPOINT® Endoscopic Fluorescence Imaging System

## Abstract

**Introduction:**

The PINPOINT® Endoscopic Fluorescence Imaging System (Novadaq, Mississauga, Canada) allows surgeons to visualize the bile ducts during laparoscopic cholecystectomy. Surgeons can continue operation while confirming the bile ducts’ fluorescence with a bright‐field/color image. However, strong fluorescence of the liver can interfere with the surgery. Here, we investigated the optimal timing of indocyanine green administration to allow fluorescent cholangiography to be performed without interference from the liver fluorescence.

**Methods:**

A total of 72 patients who underwent laparoscopic cholecystectomy were included in this study. The timing of indocyanine green administration was set immediately before surgery and at 3, 6, 9, 12, 15, 18, and 24 h before surgery. The luminance intensity ratios of gallbladder/liver, cystic duct/liver, and common bile duct/liver were measured using the ImageJ software (National Institutes of Health, Bethesda, USA). Visibility of the gallbladder and bile ducts was classified into three categories (grades A, B, and C) based on the degree of visibility in contrast to the liver.

**Results:**

The luminance intensity ratio for the gallbladder/liver, cystic duct/liver, and common bile duct/liver was ≥1 in the 15‐, 18‐, and 24‐h groups. The proportion of cases in which evaluators classified the visibility of the gallbladder and bile ducts as grade A (best visibility) reached a peak in the 15‐h group and decreased thereafter.

**Conclusions:**

In the present study, the optimal timing of indocyanine green administration for fluorescent cholangiography during laparoscopic cholecystectomy using the PINPOINT Endoscopic Fluorescence Imaging System was 15 h before surgery.

## Introduction

Laparoscopic cholecystectomy is widely accepted as an effective treatment for benign gallbladder disease, but bile duct injury remains a serious complication, with an incidence of 0.3%–0.7% 1, 2, 3, 4, 5, 6. Bile duct injury is often caused by misinterpretation of the anatomical structures rather than by insufficient technical skills 7. Several image modalities have been proposed for intraoperative visualization of the bile duct system, including routine use of intraoperative X‐ray cholangiography 8, 9, 10. However, opinions are divided on the effectiveness of intraoperative X‐ray cholangiography to avoid bile duct injury 11, 12, 13, 14.

Intraoperative fluorescent cholangiography is a novel imaging technique. Indocyanine green (ICG) is injected intravenously to enhance illumination of the bile duct structures with near‐infrared light 15. The use of ICG helps to reduce bile duct complications.

Conventional ICG fluorescence laparoscopes produce black‐and‐white images, with only fluorescence shown in color, but no full‐color systems were available in the past. In contrast, the novel PINPOINT® Endoscopic Fluorescence Imaging System (Novadaq, Mississauga, Canada) allows the simultaneous display of multiple color and fluorescence perioperative cholangiography images 16. However, during laparoscopic cholecystectomy using a conventional ICG administration method, liver fluorescence is strong, making it difficult to perform the surgery using a fluorescence screen.

We previously reported on a laparoscopic cholecystectomy performed using the PINPOINT system 17. In this case report, ICG was administered 18 h before surgery, and ICG fluorescent cholangiography was performed without interference from liver fluorescence. However, it is necessary to determine the optimal time for ICG administration before the operation.

In the present study, we investigated the optimal timing of ICG administration for fluorescent cholangiography during laparoscopic cholecystectomy using the PINPOINT Endoscopic Fluorescence Imaging System.

## Materials and Methods

### Patients

This study was conducted in accordance with the Declaration of Helsinki and approved (approval number 13‐B‐60) by the Research Ethics Committee at the International University of Health and Welfare (Nasushiobara, Japan).

A total of 72 patients who underwent laparoscopic cholecystectomy between September 2014 to August 2016 gave consent to be included in this study. We excluded patients with a history of abdominal surgery for acute cholecystitis (*n* = 4) and patients with hepatic cirrhosis (*n* = 2). Patients with acute gallbladder inflammation or abdominal surgery history were excluded because of the high probability of intra‐abdominal adhesions, which could affect surgical outcomes. In cases with liver dysfunction such as liver cirrhosis, there is a delay of excretion of ICG, so the contrast state at each administration may not be consistent with that of a patient without liver function abnormality. Therefore, we judged patients with liver dysfunction to not be suitable for this study.

Before surgery, 25‐mg ICG was administered intravenously 17. Administration occurred immediately before surgery (0‐h group) or several hours before, at 3 h (3‐h group), 6 h (6‐h group), 9 h (9‐h group), 12 h (12‐h group), 15 h (15‐h group), 18 h (18‐h group), or 24 h (24‐h group) before surgery. The patients were randomly assigned to an ICG administration time based on the order in which they were entered into the study.

### Assessments of fluorescent cholangiography

**Figure 1:**
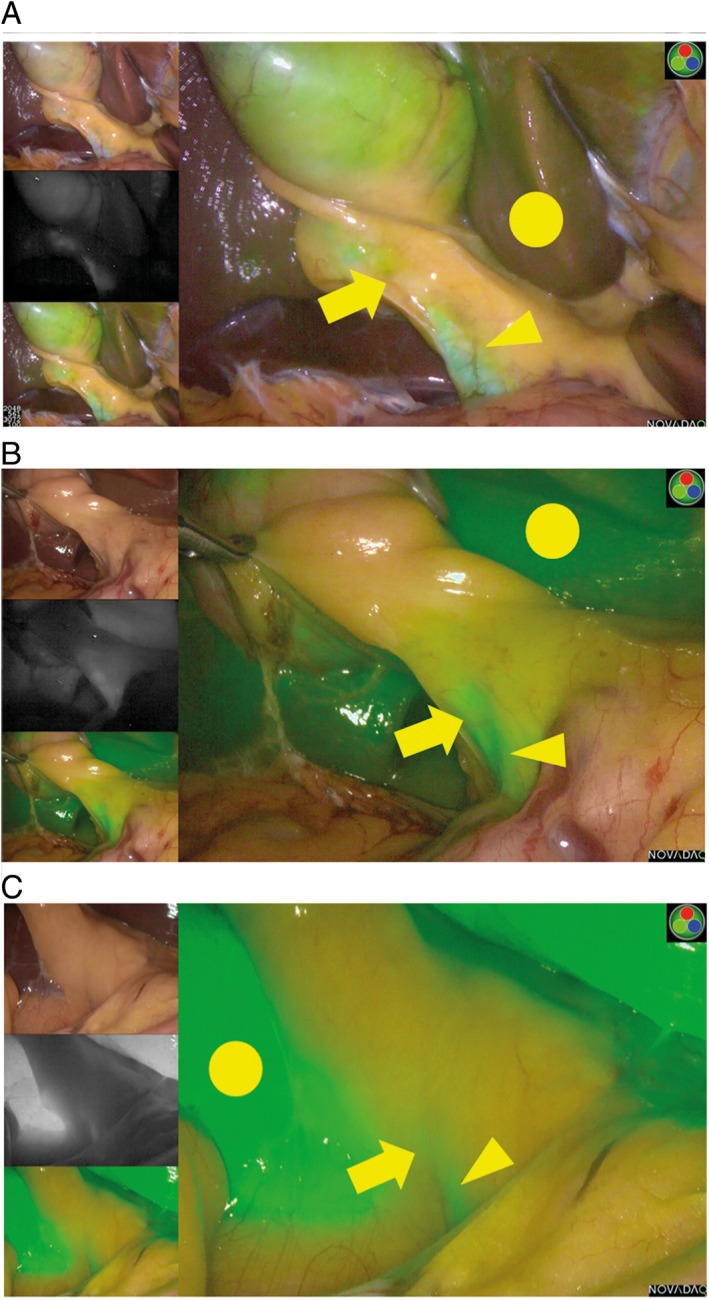
Representative images showing grades of visibility of the cystic duct (arrow), common bile duct (arrowhead), and liver (circle) in contrast to the liver. (a) Grade A, (b) grade B, and (c) grade C.

Contrast between the liver and the gallbladder, cystic duct, and common bile duct (CBD) and the visibility of the liver and bile duct were evaluated. Still images were captured on the dark‐field/black‐and‐white screen, and the luminance intensities of the liver, gallbladder, cystic duct, and CBD were measured using the ImageJ image‐processing program (http://imagej.nih.gov/ij/download.html; National Institutes of Health, Bethesda, USA) 18, 19, 20, 21. Median values of the luminance intensity ratio of the gallbladder/liver, cystic duct/liver, and CBD/liver were calculated. Visibility of the gallbladder and the bile ducts was assessed by three evaluators. (Each evaluator was a board‐certified surgeon of the Japan Surgical Society who had performed more than 100 cases of cholecystectomy.) The evaluators were blinded to the timing of the ICG administration and reviewed surgical images and photographs of the screen in which the liver, gallbladder, and bile duct were visible. We classified the visibility as grade A when the contrast of the liver was weak but the contrast of the biliary tract was strong (Figure 1a). Visibility was classified as grade C when both the contrast of the liver and the contrast of the biliary tract were strong (Figure 1c). Intermediate visibility was classified as grade B (Figure 1b). These criteria were given to the three evaluators for their individual evaluations.

### Near‐infrared imaging system

The PINPOINT system was used for fluorescent cholangiography during elective laparoscopic cholecystectomy.

To enable imaging in both high‐definition white‐light and near‐infrared fluorescence modes, the PINPOINT laparoscopes have particular features that are not available on conventional white‐light‐only laparoscopes. For example, unlike conventional laparoscopes, the PINPOINT laparoscopes are equipped with a specialized anti‐reflection coating on their optical surfaces. This enables the laparoscopes to transmit light in both the visible spectrum and the near‐infrared spectrum. The PINPOINT laparoscopes are also equipped with an excitation light rejection filter, which removes any reflected fluorescence excitation light from the image signal when the laparoscopes are used for near‐infrared fluorescence imaging. Furthermore, the PINPOINT laparoscopes are unique, even among other currently available near‐infrared light‐transmitting laparoscopes, in that only the PINPOINT laparoscopes deliver high‐definition white‐light and ICG fluorescence images projected onto the same image plane with the same magnification. This particular feature enables the PINPOINT system to acquire and simultaneously display near‐infrared fluorescence images overlaid on high‐definition white‐light in real time 22.

### Surgical technique

Because our institution is a teaching hospital, a senior resident (i.e. a physician less than 2 years after completion of senior residency) performed laparoscopic cholecystectomy.

Laparoscopic cholecystectomy was performed via four ports using PINPOINT. First, a mini‐laparotomy was performed in the lower abdomen, and a 12‐mm trocar was inserted. After CO_2_ insufflation (intra‐abdominal pressure of 10 mmHg), a 45° oblique‐viewing endoscope was inserted. In addition, under laparoscopic visualization, a 12‐mm trocar was inserted approximately 5 cm caudal to the epigastric region, a 5‐mm trocar was inserted along the midclavicular line under the right costal arch, and a 5‐mm trocar was inserted along the anterior axillary line under the right costal arch. With PINPOINT, we identified the gallbladder, cystic duct, and CBD. First, with the approach to the cystic duct, the peripheral tissue was detached, and the critical view of safety proposed by Strasberg was verified before the cystic duct was sealed using endoscopic clips 23. Subsequently, the cystic artery was sealed using endoscopic clips. The gallbladder was detached and removed using a collection bag. Before the operation concluded, the abdominal cavity was irrigated with saline to confirm the absence of bleeding or bile leakage.

Postoperative management was based on an established clinical pathway, and in the absence of perioperative complications, discharge was permitted on the third postoperative day.

### Statistical analysis

Analysis was performed with STATA® software version 13 (StataCorp, College Station, USA). The Kruskal–Wallis equality‐of‐populations rank test and the two‐sample Wilcoxon rank‐sum (Mann–Whitney) test were performed to compare the gallbladder/liver, cystic duct/liver, and CBD/liver luminance ratios for the different ICG administration times.

## Results

Baseline patient characteristics are shown in Table 1. There were no significant differences between the groups. No adverse events due to ICG administration were observed. There were no significant differences in surgical results between the groups (Table 2).

**Table 1:** Patient characteristics

	Hours before operation							
	0 (*n* = 9)	3 (*n* = 11)	6 (*n* = 9)	9 (*n* = 9)	12 (*n* = 9)	15 (*n* = 9)	18 (*n* = 8)	24 (*n* = 8)
Age (years)†	63.0 ± 19.9	67.0 ± 14.6	58.0 ± 8.0	60.0 ± 10.6	58.0 ± 10.3	65.0 ± 8.9	72.0 ± 15.7	62.5 ± 15.0
Men/women (*n*)	7/2	3/8	3/6	5/4	7/2	6/3	7/1	7/1
BMI†	22.8 ± 3.5	21.0 ± 2.8	21.8 ± 4.2	24.3 ± 5.1	26.8 ± 1.8	23.7 ± 3.5	24.0 ± 3.1	23.6 ± 2.4
Indication for cholecystectomy (*n*)								
Cholecystolithiasis	8	10	7	7	9	9	8	6
Gallbladder polyps	1	0	1	1	0	0	0	1
Adenomyomatosis	0	0	0	1	0	0	0	1
Chronic cholecystitis	0	1	1	0	0	0	0	0

† Data are presented as mean ± SD.

BMI, Body mass index.

**Table 2:** Operative outcomes

	Hours before operation							
	0 (*n* = 9)	3 (*n* = 11)	6 (*n* = 9)	9 (*n* = 9)	12 (*n* = 9)	15 (*n* = 9)	18 (*n* = 8)	24 (*n* = 8)
Operative time (min)†	98.0 ± 31.7	90.0 ± 29.6	71.0 ± 35.1	62.0 ± 22.3	85.0 ± 30.0	80.0 ± 32.0	99.5 ± 29.0	69.0 ± 27.8
Blood loss (mL)†	5.0 ± 15.0	5.0 ± 22.9	5.0 ± 335.7	5.0 ± 21.9	5.0 ± 15.0	5.0 ± 35.9	5.0 ± 17.4	5.0 ± 8.9
Intraoperative complications (*n*)	0	0	0	0	0	0	0	0
Postoperative complications (*n*)	0	0	1	0	0	0	0	1
Open conversions (*n*)	0	0	1	0	0	0	1	0
Postoperative hospital stay (days)†	3.0 ± 0.3	3.0 ± 0.6	3.0 ± 3.3	3.0 ± 0.4	3.0 ± 0.3	3.0 ± 0.3	3.0 ± 1.4	3.0 ± 2.6

† Data are presented as mean ± SD.

### Gallbladder/liver luminance intensity ratio

**Figure 2:**
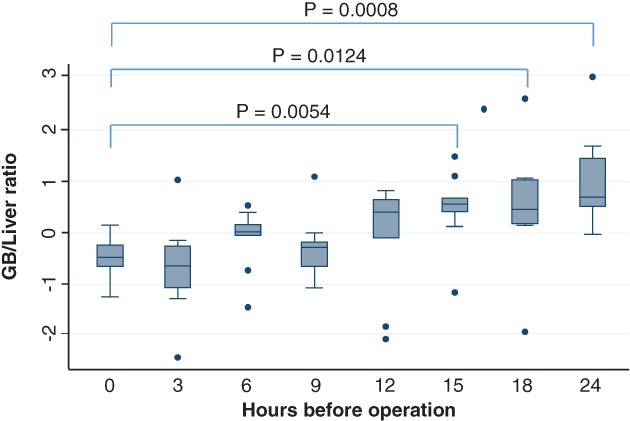
Luminance intensity ratio of the gallbladder (GB)/liver and timing of indocyanine green administration. A significant difference was observed between the 0‐h group and the 15‐h, 18‐h, and 24‐h groups.

In the 6‐h group, the gallbladder/liver luminance intensity ratio exceeded 1, but it decreased to less than 1 in the 9‐h group. In addition, it exceeded 1 in the 12‐, 15‐, 18‐, and 24‐h groups. A significant difference was observed between the 0‐h group and the 15‐, 18‐, and 24‐h groups (Figure 2).

### C**ystic duct/liver luminance intensity ratio**

**Figure 3:**
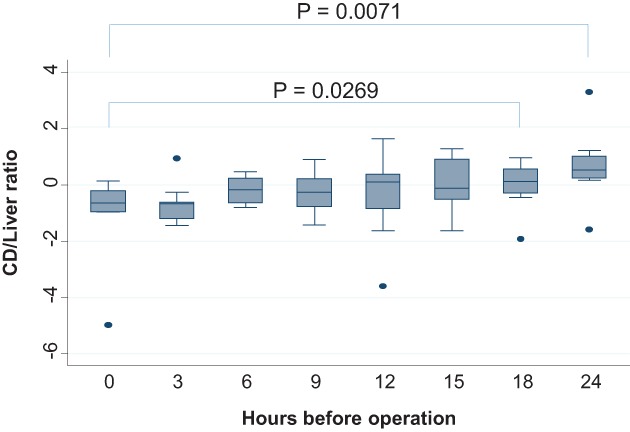
Luminance intensity ratio of the cystic duct (CD)/liver and timing of indocyanine green administration. A significant difference was observed between the 0‐h group and the 18‐h and 24‐h groups.

In the 12‐, 15‐, 18‐, and 24‐h groups, the cystic duct/liver luminance intensity ratio exceeded 1. A significant difference was observed between the 0‐h group and the 18‐h group, as well as between the 0‐h group and the 24‐h group (Figure 3).

### CBD**/liver luminance intensity ratio**

**Figure 4:**
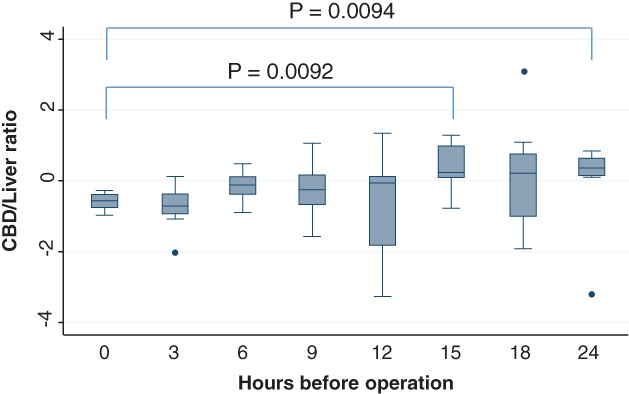
Luminance intensity ratio of the common bile duct (CBD)/liver and timing of indocyanine green administration. The luminance intensity ratio was the highest in the 15‐h group, and the difference between the 0‐h group and the 15‐h and 24‐h groups was significant.

In the 12‐, 15‐, 18‐, and 24‐h groups, the CBD/liver luminance intensity ratio exceeded 1. The luminance intensity ratio was the highest in the 15‐h group, and the difference between it and the 0‐h group was significant (Figure 4). Also, there was a significant difference between the 0‐h group and the 24‐h group.

### Visibility of the gallbladder and bile ducts

**Figure 5:**
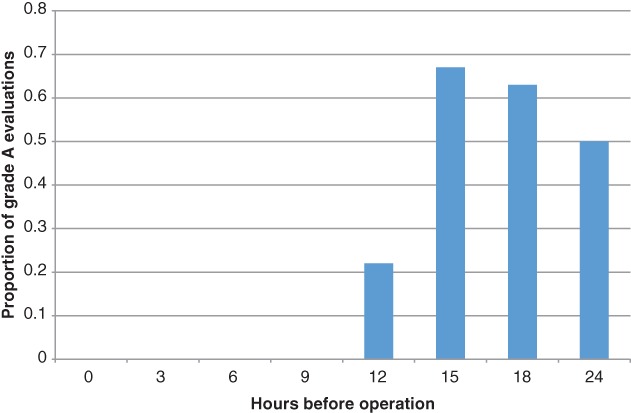
The proportion of cases in which two or more evaluators classified the visibility of the gallbladder and bile ducts as grade A first increased with the 12‐h group, reached a peak in the 15‐h group, and decreased thereafter.

The proportion of cases in which two or more evaluators classified the visibility of the gallbladder and bile ducts as grade A first increased with the 12‐h group, reached a peak in the 15‐h group, and decreased thereafter (Figure 5).

## Discussion

In the current study, intraoperative ICG fluorescent cholangiography was performed to increase visibility of the bile ducts during laparoscopic cholecystectomy. We investigated the optimum timing of ICG administration for strong fluorescence intensity of the biliary tract and weak fluorescence intensity of the liver.

ICG can be administered intravenously or intra‐arterially. It absorbs light in the near‐infrared region at 805 nm and emits fluorescence at a slightly longer wavelength of 830 nm. When injected intravenously, ICG rapidly and extensively binds to plasma proteins and is confined to the intravascular compartment with minimal leakage into the interstitium 22. Although the fluorescence of ICG cannot be observed by the naked eye, it is not significantly influenced by the absorbance of hemoglobin or water. Therefore, using a charge‐coupled device camera with sensitivity in the near‐infrared region makes it possible to visualize a structure containing ICG through connective tissue with a thickness of 5–10 mm. Because of ICG's fluorescence properties and the fact that it is excreted almost exclusively into bile, it is possible to identify and visualize the biliary tract with PINPOINT.

Conventional ICG fluoroscopy laparoscopes produce dark‐field, black‐and‐white images with no bright field or color. The bright‐field/color fluorescence camera of the HyperEye Medical System® (Mizuho, Tokyo, Japan) has been used in gastroenterological surgeries to visualize sentinel lymph nodes and blood flow 24, 25.

The PINPOINT laparoscopic system uses bright‐field/color fluorescence, and the cystic duct and CBD can be visualized after ICG administration. Moreover, because the system allows toggling between normal color and contrast screens, surgery can be performed seamlessly without changing the laparoscope.

Laparoscopic cholecystectomy is performed behind the liver, and visibility improves with lower liver luminance. If the brightness of the gallbladder, cystic duct, and CBD is higher than the brightness of the liver, the contrast allows the operation to be performed safely.

In 2010, Ishizawa *et al.* and Aoki *et al.* separately published studies on ICG cholangiography 26, 27. In their studies, Aoki *et al.* administered ICG at 12.5 mg/body, and Ishizawa *et al.* did so at 2.5 mg/body. Kono *et al.* reported that the time from ICG administration to observation with a fluorescence camera was significantly longer in patients in whom the cystic duct–CBD junction could be identified using fluorescence before dissection of Calot's triangle than in patients in whom the bile duct anatomy could not be delineated using fluorescence (median, 90 vs 47 min; *P*  <  0.01) 28. They showed that the quality of cholangiography changes according to the timing of ICG administration. In 2014, Verbeek *et al.* published paper on the dosage and timing of ICG administration 29. They administered 5 mg at 30 min preoperatively, 10 mg at 30 min preoperatively, 10 mg at 24 h preoperatively, or 20 mg at 24 h preoperatively. They showed that a dose of 10‐mg ICG injected 24 h before surgery provided a significantly lower liver background signal while the CBD signal remained stable. This result is noteworthy, but several issues need to be addressed. Verbeek *et al.* examined two timings only. To administer ICG 24 h before surgery, patients may need to be hospitalized 2 days before surgery or come to hospital more than 24 h before surgery. Also, timings between 30 min and 24 h remained unexamined. In their study, the results of CBD‐to‐liver ratio varied widely, with a range of 1.1–6.2 (median, 2.3) in the group who received 10 mg 24 h before surgery. The variance was smaller in the group who received 20 mg 24 h before surgery, with a range of 1.6–2.9 (median, 1.7). In our current study, we examined eight timings with a fixed maximum dosage (25 mg/body). In 2016, while our current study was being conducted, Zarrinpar *et al.* reported on the dosage (0.02–0.25 mg/kg) and the timings (10–180 min) of ICG administration and concluded that a dose of 0.25 mg/kg administered at least 45 min before visualization facilitates intraoperative anatomical identification 30. However, the data regarding administration between 180 min to 24 h remain unknown.

In the present study, the gallbladder/liver, cystic duct/liver, and CBD/liver contrast‐to‐background ratio was 1 or higher in the 15‐, 18‐, and 24‐h groups, and the best time for ICG administration seems to be 15 or 18 h preoperatively.

The proportion of cases in which two or more evaluators classified the visibility of the gallbladder and bile ducts as grade A first increased with the 12‐h group, reached a peak in the 15‐h group, and decreased thereafter. Our results indicated that ICG is best administered at 15 h.

Based on contrast‐to‐background ratios, the timing of preoperative administration was best at 15 h before surgery. For the visibility, the timing of preoperative administration was also best at 15 h before surgery.

According to our evaluations of luminance and visibility, the optimal timing of ICG administration for fluorescent cholangiography during laparoscopic cholecystectomy using the PINPOINT Endoscopic Fluorescence Imaging System was 15 h preoperatively.
